# Tyr^682^ in the Intracellular Domain of APP Regulates Amyloidogenic APP Processing *In Vivo*

**DOI:** 10.1371/journal.pone.0015503

**Published:** 2010-11-16

**Authors:** Alessia P. M. Barbagallo, Richard Weldon, Robert Tamayev, Dawang Zhou, Luca Giliberto, Oded Foreman, Luciano D'Adamio

**Affiliations:** 1 Department of Microbiology and Immunology, Albert Einstein College of Medicine, Bronx, New York, United States of America; 2 Department of Laboratory Animal Health, The Jackson Laboratory, Bar Harbor, Maine, United States of America; 3 The Litwin-Zucker Research Center for the Study of Alzheimer's Disease, The Feinstein Institute for Medical Research, North Shore – LIJ, Manhasset, New York, United States of America; Biological Research Center of the Hungarian Academy of Sciences, Institute of Biophysics, Hungary

## Abstract

**Background:**

The pathogenesis of Alzheimer's disease is attributed to misfolding of Amyloid-β (Aβ) peptides. Aβ is generated during amyloidogenic processing of Aβ-precursor protein (APP). Another characteristic of the AD brain is increased phosphorylation of APP amino acid Tyr^682^. Tyr^682^ is part of the Y^682^ENPTY^687^ motif, a docking site for interaction with cytosolic proteins that regulate APP metabolism and signaling. For example, normal Aβ generation and secretion are dependent upon Tyr^682^ *in vitro*. However, physiological functions of Tyr^682^ are unknown.

**Methodology/Principal Findings:**

To this end, we have generated an APP Y682G knock-in (KI) mouse to help dissect the role of APP Tyr^682^ *in vivo*. We have analyzed proteolytic products from both the amyloidogenic and non-amyloidogenic processing of APP and measure a profound shift towards non-amyloidogenic processing in APP KI mice. In addition, we demonstrate the essential nature of amino acid Tyr^682^ for the APP/Fe65 interaction *in vivo*.

**Conclusions/Significance:**

Together, these observations point to an essential role of APP intracellular domain for normal APP processing and function *in vivo*, and provide rationale for further studies into physiological functions associated with this important phosphorylation site.

## Introduction

The most common form of dementia in the world is Alzheimer's disease (AD), affecting about 1% of the human population by aged 65, and rising to 35–40% after age 85. Evidence points to a key role for misfolded amyloidogenic Aβ peptides in the pathogenesis of AD (amyloid cascade hypothesis). The accumulation of Aβ as plaques in the hippocampus and other brain regions is a key characteristic of AD pathology [1], [2].

Aβ peptides are generated during amyloidogenic processing of Aβ-precursor protein (APP). When APP is cleaved by β-secretase, the soluble ectodomain (sAPPβ) is released extracellularly whilst the 99 amino acid C-terminal fragment (C99) remains membrane bound. In a second proteolytic event, C99 is cleaved by the γ-secretase. Two peptides are released, Aβ peptide consisting of either 40 or 42 amino acids (Aβ40 and Aβ42, respectively) and an intracellular product (AID or AICD), which regulates apoptosis [3] and transcription [4]. An alternative, non-amyloidogenic pathway also exists. In this pathway APP is cleaved by α-secretase in the Aβ sequence producing the soluble ectodomain (sAPPα) and the membrane bound 83 amino acid C-terminal fragment (C83). C83 is also further cleaved by the γ-secretase into the P3 and AID peptides.

The degree to which APP function plays a role in the pathogenesis of AD is unclear but changes in the apoptotic and axonal activities of APP may underlie some aspects of AD pathology [5], [6]. However, until the *in vivo* functions of APP are better understood this will remain a crucial question. APP null mice have retarded neuron development, reduced hippocampal neuron viability, diminished grip strength, locomotor activity and postnatal growth [7], but can be normalized by over expression of the sAPP-α ectodomain [8]. However, since the essential functions of APP are compensated for by homologues APLP1 and APLP2, the physiological significance of the short intracellular C-terminal domain remains relatively unexplored *in vivo*.

The ∼50 amino acids long APP intracellular region contains seven residues that can be phosphorylated andseveral of these amino acids are hyperphosphorylated in human AD brain. However,it remains unclear whether this is a cause or a consequence of neurodegeneration. One of these sites of particular functional significance is Tyr^682^, the phosphorylation of which is increased in AD patients [9], especially in vasculature tissue of the brain [10]. This residue forms an essential part of the evolutionarily conserved, canonical endocytic Y^682^ENPTY^687^ motif. Compromised endocytosis of APP is shown to significantly decrease amyloidogenic processing and Aβ secretion [11]. This motif is also a docking site for cytosolic proteins, such as Fe65, that regulate APP metabolism and signaling [12]. Phosphorylation of Tyr^682^ promotes interaction of Src-Homology 2 domain (SH2) while it reduces interaction with a subset of proteins containing a Phospho-Tyrosine-Binding (PTB) domain *in vitro* [13], [14]. The potential role of Tyr^682^ phosphorylation state as a “biochemical switch” to change the molecular composition of APP complexes is an intriguing possibility. In the AD brain, a possible pathological role for augmented APP phosphorylation on Tyr^682^ needs further exploration. To begin to specifically dissect the functional role of APP intracellular domain *in vivo* we have generated APP KI mice with a mutation in amino acid Tyr^682^. Here we describe the generation and initial characterization of APP KI mouse with mutation of Tyr^682^mutation of Tyr^682^.

## Materials and Methods

### Ethics Statement

Mice were handled according to the Ethical Guidelines for Treatment of Laboratory Animals of Albert Einstein College of Medicine. The procedures were described and approved in animal protocol number 20040707

### Generation of APP Y682G and T668A mutant Mice

A 7.0-Kb genomic fragment containing exon 16 from C57BL/6 BAC DNA (RP23-99P18) was amplified by PCR with the following primers:

Fwd: 5′-aaaaGGTACCagtatctcttgtcctcacaatg-3′;

Rev: 5′-aaaaCCGCGGtaggtcccaaggcta-3′.

This fragment flanked by KpnI and SacII sites was cloned into pBS (pBS-EX16), and used as a template for subsequent cloning. Two nucleotide mutations were introduced into pBS-EX16 vector by site directed mutagenesis PCR. Firstly a SmaI/XmaI restriction site (CCC GGA → CCC GGG) was created right in front of exon 16 by using the following primers:

Fwd: 5′-ctattttaaacccggatctctgtacctgctttc-3′;

Rev: 5′-gaaagcaggtacagagatccgggtttaaaatag-3′.

This new restriction site was used to verify the targeted clone. Furthermore the nucleotide change from either ACC to GCC or TAT to GGA in exon 16 generated the corresponding amino acid mutation T668A or Y682G, respectively. The following primers were used for this mutation: for T66A mutation,

Fwd, 5′- TCGACGCCGCCGTGGCCCCAGAGGAGCGCCATCT -3′;

Rev, 5′- AGATGGCGCTCCTCTGGGGCCACGGCGGCGTCGA -3′;

And for Y682G mutation,

Fwd, 5′-tgcagcagaacggaggagagaatccaact -3′;

Rev, 5′- agttggattctctcctccgttctgctgca-3′.

The 1.3 Kb Hind III-Sal I left arm and 2.4 Kb NotI-SacII right arm were amplified from above mutated pBS-Ex16 by using the following primers. For right arm:

Fwd, 5′- aaaaaaagcttcaatggccatgaagga - 3′;

Rev, 5′-aaaaagtcgaccaggtatctgctgccat-3′;

For left arm:

Fwd, 5′- aaaaagcggccgcggccccacaaagcggagt -3′;

Rev, 5′- aaaaaccgcggtggcgcatgctgcag- 3′.

The left arm contains SmaI/XmaI and either T668A or Y682G mutations. Subsequently, the left arm and right arm were inserted into a Soriano PGK-Neo-dTA vector. The resulting construct was thus:

--- dTA cassette-Left Arm-LoxP1-PGK-Neomycin cassette-LoxP2-Right Arm ---

The resulting construct was linearized with KpnI and purified prior to injection in ES cells strain 129 by electroporation. ES culture was performed on feeder layer, and further electroporation and handling was also performed according to the methodology employed at Dept of Cell Biology, Albert Einstein College of Medicine, and according to Wakayama et al. In particular, after electroporation, ES cells were re-plated in 55 cm^2^ dishes and grown until visible clones appeared. Clones were then picked and transferred to 96 well plates in triplicates. Triplicates were either screened by PCR or frozen for subsequent use and further analysis.

Homologous recombinants were selected with G418 (200 µg/ml) and dTA exclusion. Injection of the two Y682G mutant targeted ES cell clones into C57BL/6J blastocysts was performed at the Albert Einstein College of Medicine gene-targeting facility, according to the facility protocol.

### PCR Analysis

The PCR screening was performed using the Expand Long Template PCR System (Roche-applied-Science) with Betaine, according to the manufacturer instructions. PCR analysis of recombinant ES cells and mice was conducted with the following primers and digestion strategies to identify the correct recombinant clones and strains:

ES cells:

Left arm:

Genomic primer: 5′-CAGAAGGAAATGTCCCAGGA-3′

Neo cassette primer: 5′-CTTCTAGTTGCCAGCCATCTG-3′

Product: 1687 bp

Right arm:

Genomic primer: 5′-GGATCTCACCCTGTTTTCCA-3′

Neo cassette primer: 5′- TGCACGAGACTAGTGAGACGTG-3′

Product: 3306 bp

Amplification of the right arm and digestion:

Genomic upstream primer: 5′-CTACAGAGATAAATGTACTTCG-3′

Genomic downstream primer: 5′-GGATCTCACCCTGTTTTCCA-3′

Product: 3200 bp  = wild type without SmaI/XmaI restriction site→3200 bp

Product: 3200 bp  =  mutant alle with SmaI/XmaI restriction site; SmaI digestion→2800 bp +400 bp

Mice genotyping:

Fw primer: 5′-ATGGCACCACCCACAATAGG-3′

Rev primer: 5′-CCTAGCAACTGGTAACAGTGC-3′

Product: 2027 bp with Neo cassette

Product: 332 bp without Neo cassette

Product: 194 bp wild type

PCR products were digested to ascertain that the targeted sequence was correctly inserted in the genomic DNA.

### Southern Blot Analysis

Twenty µg of genomic DNA was digested with BamHI overnight, run on a 1% TAE agarose gel and transferred on a Hybond-N+ membrane (Amersham).

The probe was prepared by PCR from a BAC clone (RP23-99P18) with the following primers:

-Left arm:

Fw: 5′-GGATCCCACCTCGTGCAGATG -3′

Rev: 5′-GGAGTAATTCAGGTGGAG -3′

Probe size: 232 bp

-Right arm:

Fw: 5′-actgggtggaaacacctgag-3′

Rev: 5′-gagagaggagcctgcagaga-3′

Probe size: 542 bp.

One µg of PCR probe was labelled with 5 µL of 32P-dCTP (3000 Ci/mmol, ICN) and purified through a Push Column (Stratagene) according to the manufacturer's protocol. Membranes, containing the cleaved genomic DNA, were hybridized at 65°C and subsequently washed 4 times in SSC buffer (Sigma). Film was exposed to the hybridised membranes at −80°C and then developed.

### Immunoblot analysis

Whole mouse brain was dounce homogenized (1∶10 w/v) in tissue homogenization buffer (20 mM Tris-base pH 7.4, 250 mM sucrose, 1 mM EDTA, 1 mM EGTA plus protease (Roche, Complete) and phosphatase inhibitors. For detection of full length APP or APP CTF's, the lysates were spun at 1,000 g for 15 min and an equal amount whole protein homogenates were loaded for either 4–20% SDS-PAGE or 13% tris-tricine SDS-PAGE respectively and transferred onto nitrocellulose membranes for detection using AbD (Zymed). For detection of sAPPα and β (IBL antibody #27724 & #27722, respectively) an additional 45 min spin at 100,000 g was used to remove membranes prior to SDS-PAGE. Finally, in order enhance the signal for detection of both sAPPβ and APP CTF's, the nitrocellulose membranes were subject to epitope retrieval, prior to blocking, through incubation with boiling PBS-T and subsequent cooling to room temperature.

### Reverse Transcriptase-PCR and Real Time Quantitative PCR Analysis

Mouse brain mRNA was extracted with Trizol reagent (Invitrogen). Briefly, one mouse hemisphere was shock frozen, weighed and homogenized in 4 volumes of Trizol reagent with an electric dounce homogenizer, 3×30” in ice. The suspension was cleared of debris and membranes by centrifugation, and nucleic acids were separated by chloroform extraction and ethanol precipitation. The mix was applied to RNeasy columns, and RNA purified with RNeasy Protect Kit (Qiagen) according to the manufacturers' protocols, including on-column DNase digestion (Qiagen). One µg of RNA, quantified with the Nanodrop (Thermo Scientific), was reverse-transcribed to cDNA using random primers and the SuperScript III First-Strand Synthesis System for RT-PCR kit (Invitrogen). Real time PCR was based on the TaqMan technology, using 200 ng of cDNA and mouse APP, Beta Actin and beta-2-microglobulin inventoried assays (Mm01344172_m1, Mm00607939_s1 and m00437762_m1, Applied Biosystems) according to the manufacturer's protocols, in 20 µL volume and in 96well plate format. The threshold cycles (Ct) for the endogenous controls mRNA (β-actin and β-2-microglobulin) and the target (APP) signal were determined and the relative RNA quantification was calculated using the comparative DDCt method. Each experiment was conducted in triplicate. Data analysis was conducted according to Applied Biosystems references and protocols, and using student-t test.

### Neuronal cultures

Neuronal cultures were performed as described previously [15], [16] from E16-17 fetuses.

### Mouse Dermal Fibroblasts

To culture mouse dermal fibroblasts (MDFs), skin was removed from mouse tails, soaked in 70% ethanol, washed in PBS, diced into small pieces and incubated at 37°C overnight in CO_2_ incubator in DMEM containing 20% FBS, supplemented with penicillin/streptomycin and 1.6 mg/ml collagenase II. On the next day, clumps were removed by passing through a nylon mesh, and the material was centrifuged at 1000 rpm for 5 min to collect the cells. The collected cells were maintained in DMEM containing 20% FBS and penicillin/streptomycin.

### Biotinylation and streptavidin precipitation

For biotinylation experiments, MDFs were washed three times with cold PBS plus Ca^++^ and Mg^++^ (PBS-CM) and labelled for 30 min on ice in 0.5 mg/ml sulfo-NHS-SS-biotin (Pierce) dissolved in PBS-CM. Free biotinylation reagent was removed by washing three times with PBS-CM containing 0.1% BSA. The cells were lysed in the RIPA buffer. The lysates were cleared by centrifuging at 20,000 g for 10 min, and were mixed with streptavidin agarose beads (Sigma S1638). After collecting unbound lysate, the beads were washed four times with the RIPA buffer, and were boiled in 2× SDS buffer. Comparable volume of the samples were subjected to western blot.

### Synaptosomes

For *synaptosomes*, mouse brain was homogenized in H buffer [5 mM Hepes/NaOH pH 7.4, 1 mM EDTA, 1 mM EGTA, 0.32 M sucrose, plus protease (PI) and phosphatase (PhI) inhibitors] at 10% (w/v) and centrifuged at 800 g for 10 min. The supernatant (S1) was separated to supernatant (S2) and pellet (P2) by spinning at 9,200 g for 15 min. P2 was lysed on ice for 30 min in H buffer containing 35.6 mM sucrose. The lysed P2 was separated to supernatant (LS1) and pellet (LP1) by spinning at 25,000 g for 20 min. S2 was separated to supernatants (S3) and pellet (P3) by spinning at 165,000 g for 2 hrs, respectively. P3 was suspended in H buffer containing 0.32 M sucrose by sonication. Synaptosomes fractions represent: S1, postnuclear supernatant; S2, cytosol, soluble proteins and light membrane; P2, crude synaptosomal fraction; S3, soluble fraction; P3, light membrane abundant in Golgi and ER; LS1, crude synaptic soluble; LP1, synaptic membrane fraction.

### Aβ40 ELISA

DEA extraction of Aβ from brain lysates was carried out as previously described [17]. Prior to ELISA, DEA extracts were further purified with Oasis HLB sample extraction cartridges (Waters, WAT094226) to decrease background artifacts which otherwise prevent detection of endogenous wild-type Aβ40 in mice [18]. Aβ40 ELISA kit (IBL America, discontinued product) was used according to manufacturer's protocol with equal quantities of protein loaded.

### Co-immunoprecipitation

Whole mouse brain lysate was centrifuged at 9000 g for 15 min and the resulting supernatant at 100,000 g for 45 min. The supernatant was removed and the membrane pellet re-suspended overnight in IP buffer (20 mM Hepes pH 8.0, 10% glycerol, 137 mM NaCl, 0.5% Triton-X-100, 2 mM EDTA) Any remaining debris was removed with centrifugation at 9000 g for 15 min and the membrane enriched supernatant diluted to 1 mg/ml in IP buffer. 500 ug of membrane protein was used for each IP. Supernatants were pre-cleared with 30 ul protein A/G (Pierce) for 30 min. 2 µg of either monoclonal anti-APP, non-specific mouse monoclonal control, anti-Fe65 (I12) [19] or non-specific rabbit polyclonal control were added for incubation for 30 min. 20 ul protein A/G was then added for overnight incubation. Supernatant was removed and the beads were washed 4× with IP buffer. Beads were re-suspended in 1× LDS buffer with 10% BME and 1% NEM and incubated for 5 min at 95°C. Immunoblot analysis was carried out as described above with α-APP IP probed for Fe65 and α-Fe65 IP probed for full length APP (22C11 antibody, Millipore, 1∶1500).

### Pathological evaluation

Complete necropsy was performed on all mice and tissues collected were fixed in Tellyesniczky/Fekete fixative (100 ml 70% ethanol, 5 ml 37–40% formalin, 5 ml glacial acetic acid). Appropriate tissues were decalcified using 10% formic acid (Formical-2000®, Decal Chemical Corporation, Tallman, NY) or 3% hydrochloric acid (Cal-Ex®, Fisher Scientific, Fairlawn, NJ) for 24 hours. Tissues were paraffin embedded and stained with hematoxylin and eosin (H&E). A board certified veterinary pathologist with no knowledge of the genotypes analyzed the slides.

## Results

### Generation of APP Y682G and T668A Mutant Mice

**Figure 1:**
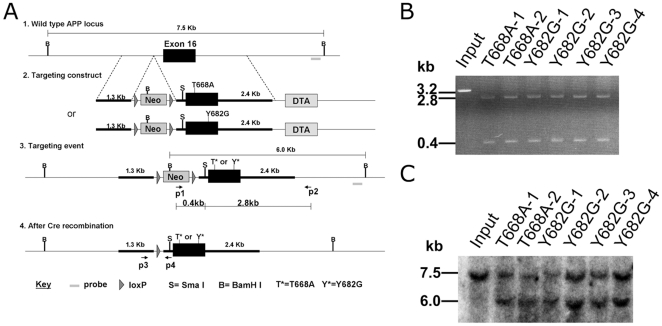
Generation of APP^YG^ mice. **A**, Schematic representation of the construct injected in 129 ES cells, showing site of APP T668A and Y682G mutation on last Exon 16, primer sites, site of Southern Blot probe, LoxP, pgk-dta and pgk-Neo sites. The bottom graphics depict the construct with and without the pgk-Neo cassette that has been removed by means of Cre recombinase. **B**, The right arm (p1–p2) PCR analysis of six positively targeted ES clones. A 3.2 Kb PCR product digested by a novel restriction site Sma I produce 0.4 Kb and 2.8 Kb fragment. **C**, Southern Blot showing a shift from the 7.5 Kb of the wild type genome to the 6.0 Kb band of two T668A and four Y682G positively knock in ES clones for the homologous recombination of the mutated allele, due to the insertion of a new BamHI site.

The targeting strategy for the generation of the APP KI mice entailed the replacement of APP exon 16 with exon 16 carrying the Y682G or T668A mutation (Fig. 1A). The vector used the floxed PGK-neo selection cassette and contains a 5′ homologous region and the negative selection cassette, PGK-dta. The 3′ homologous region introduced the T668A or Y682G mutation, BamHI and SmaI sites into the APP mouse gene. The linearized targeting vector was transfected into 129 ES cells. In the presence of the positive selection drug, G418, clones only survived if both the PGK-neo selection cassette was integrated and the PGK-dta cassette was removed by homologous recombination. ES cell clones carrying the targeting vector by random, non-homologous integration, were eliminated due to expression of diphtheria toxin.

After selection, ES cell clones carrying the proper homologous recombination and the Y682G or T668A mutant allele were identified by PCR for 3′ region (i.e. right Arm: if homologous recombination had occurred these primers would amplify a product of 3.2 Kb). Out of ∼600 screened ES clones, we found two targeted clones for T668A and four clones for Y682G mutation (Fig. 1B). Also, PCR amplification and digestion was used to check the proper insertion of the construct in the genomic DNA and the removal of the Neo cassette (not shown).

The occurrence of homologous recombination was confirmed by both sequencing and Southern blot analysis (Fig. 1C). DNA derived from individual Y682G or T668A ES clones was digested with BamHI, gel separated, blotted into a nylon membrane and hybridized with the 3′probe. The 3′ probe hybridizes with a ∼7.5 Kb fragment derived from the wild-type locus. Homologous recombination at the 3′ homologous region yields a ∼6.0 Kb fragment upon BamHI digestion due to the introduction of the BamHI site and the PGK-neo selection cassette. ES clones (T668A or Y682G) carry a wild type allele (7.5 Kb) and a recombined allele (6.0 Kb). The 7.5 Kb and 6.0 Kb bands had a similar intensityproving the ES cells selected were clonal populations. Similar results were obtained when homologous recombination at the 5′ site was assessed.

Two ES cell clones each for Y682G or T668A (129, agouti coat color), carrying the correct site-specific homologous recombination, were injected into C57BL/6J blastocysts (black coat color). The resulting chimeras with a high proportion of agouti coat color (i.e. with a high relative contribution from the injected ES cells) were backcrossed to C57BL/6J mice to obtain heterozygous Y682G/wt, which were identified by PCR and Southern analysis as described above (not shown) using tail DNA. Heterozygous mice were crossed to Meu40-Cre mice to obtain Meu40/APP Y682G/wt or Meu40/APP T668A/wt animals. Cre is a bacteriophage P1-encoded recombinase that catalyzes site-specific recombination between two 34 bp loxP recognition sites, resulting in the excision of the intervening DNA sequences. The resulting mice are named APP Y682G or APP T668A and are abbreviated to APP YG or TA respectively, where appropriate.

### Reduced β-cleavage and enhanced α-processing of APP Y682G mutant mice

**Figure 2:**
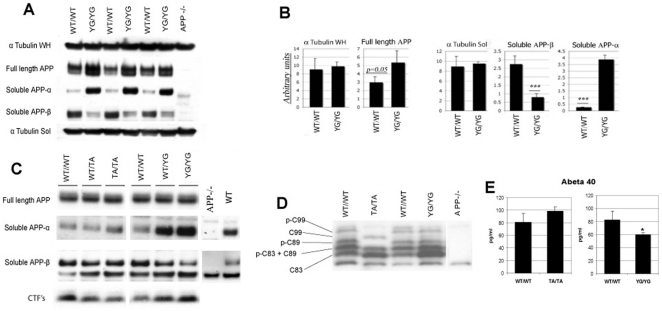
Altered APP proteolytic processing in APP^YG/YG^ mouse brain. APP^−/−^ brain was used as a negative control in each experiment. **A**, Immunoblot analysis comparing full length APP (WH  =  whole homogenates), sAPP-α, sAPP-b and tubulin levels between APP^wt/wt^ APP^YG/YG^ mice (n = 3). **B**, Quantitative analysis of panel A normalized to tubulin (_***_  =  p<0.01). **C**, WB analysis of APP^wt/wt^, APP^wt/TA^, APP^TA/TA^, APP^wt/YG^ and APP^YG/YG^ brain lysate showing full length APP, sAPP-α and sAPP-β. **D**, WB analysis of APP CTF's using a tris-tricine gel, and comparing APP^wt/wt^ with APP^TA/TA^ and APP^YG/YG^. Five specific species representing C83, C89, C99 and their respective phosphorylated forms can be identified, noting that p-C83 and C89 overlap. Bands at the very top and bottom are non-specific. **E**, Aβ4ELISA comparing APP^wt/wt^ with APP^TA/TA^ (n = 3) and APP^wt/wt^ with APP^YG/YG^ (n = 4). APP^−/−^ mice were also used to validate specificity of the assay (data not shown).

Previous studies have shown an important role for Tyr^682^ in shifting APP toward the amyloidogenic (β-processing) rather than the non-amyloidogenic (α-processing) pathway *in vitro* [11]. Using the models described above we established whether this is true *in vivo*. Rates of α and β processing are reflected by the relative abundance of the products of these two pathways, sAPP-α and sAPP-β, respectively. Using immunoblot analysis an approximately 15-fold increase in sAPP-α in conjunction with a 3.5 fold decrease in sAPP-β was detected in brain tissue from APP^YG/YG^ mice compared to APP^wt/wt^ controls (Fig. 2A & 2B). Importantly, no differences in sAPP-α and sAPP-β between APP^TA/TA^, APP^TA/wt^ and APP^wt/wt^ mice (Fig. 2C) was detected thus demonstrating the highly specific role of Tyr^682^ in modulating entry of APP into the amyloidogenic pathway *in vivo*.

**Figure 3:**
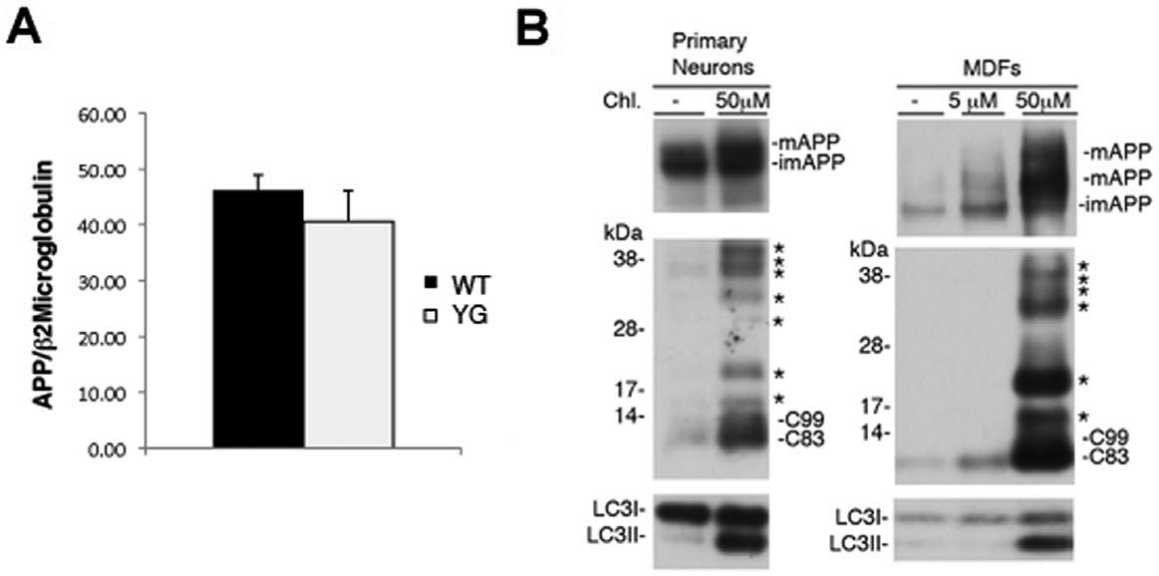
Normal expression of the mutant *APP* allele. **A**, The genetic manipulation of the *APP* gene locus in *APP^YG/YG^* mice does not affect transcription/splicing of *APP* since mRNA *APP* levels are comparable to those transcribed in age-matched *APP^WT/WT^* mice (analogous data were obtained using Beta Actin as housekeeping gene). **B**, APP and APP-derived CTFs are processed by the lysosomes. Inhibition of lysosomal activity by chloroquine (Chl.) results in accumulation of APP and APP derived fragments. The asterisks indicate APP-CTFs that are derived by cleavages in the ectodomain NH_2_-terminal to the site processed by BACE1. The effectiveness of Chl. in inhibiting lysosomal degradation (inhibition occurs at 50 µM concentration but not at 5 mM) is confirmed by the accumulation on LC3II [78]. The antibody against LC3 is from Cell Signaling.

**Figure 4:**
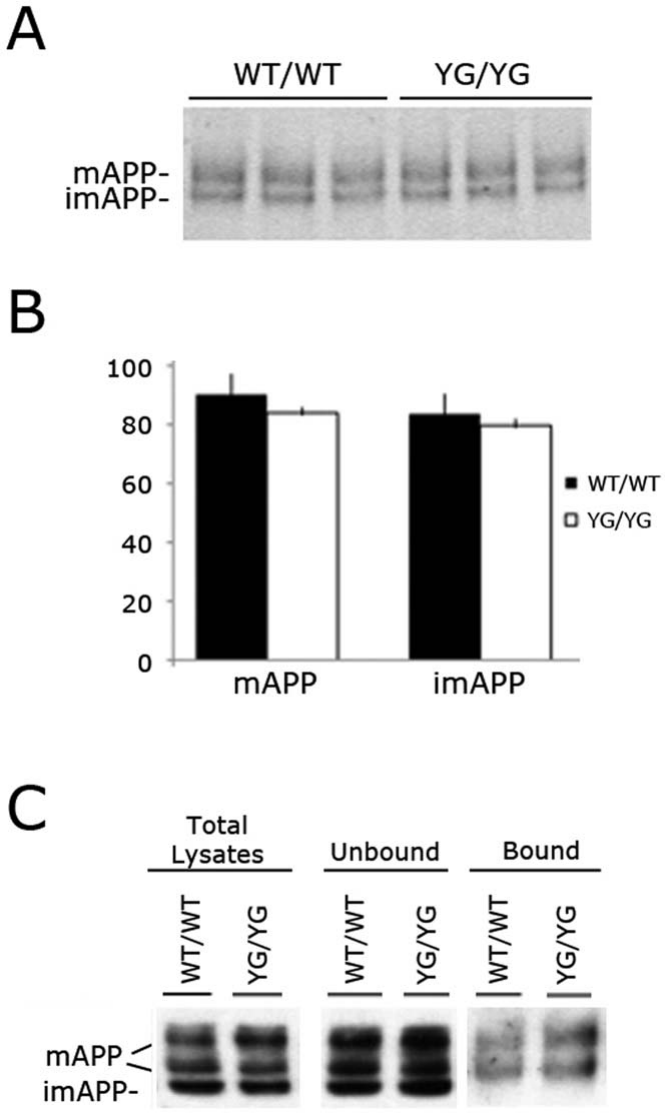
Maturation and membrane levels of APP are unaffected by the YG mutation. **A**, Immunoblot analysis comparing imAPP and mAPP between APP^wt/wt^ and APP^YG/YG^ mice (n = 3). **B**, Quantitative analysis of panel A shows no differences in mAPP and imAPP levels between the two genotypes. **C**, Biotynilation experiment in APP^wt/wt^ and APP^YG/YG^ MDFs shows comparable levels of im and mAPP, as well as cell membrane levels of mAPP.

**Figure 5:**
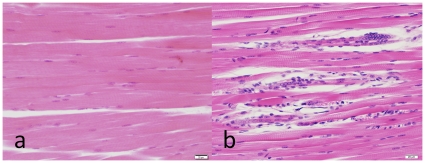
Alterations in quadriceps muscle from two APP^YG/YG^ mice. **A**, normal striated muscle. **B**, muscle fibers undergoing regeneration.

An analysis of APP COOH-terminal fragments (Fig. 2D) shows that C83, which is formed in conjunction with sAPP-α, is greatly increased in APP^YG/YG^ mice over APP^wt/wt^ control, consistent with an increase in non-amyloidogenic processing. The level of C99, which is formed in conjunction with sAPP-β, does not change appreciably. Interestingly, the level of the phosphorylated APP COOH-terminal fragments, p-C99, p-C89 and p-C83, were unchanged in APP^YG/YG^ but, consistent with observations by Sano, Y. et al. [20], were below detectable levels in APP^TA/TA^ mice. This indicates that the steady-state phosphorylation of APP is predominantly located on Thr^668^ in the brain and that phosphorylation of Tyr^682^ is highly regulated (perhaps by signaling mechanisms) and may have a short half-life. Also, it shows that the Y to G mutation has not grossly altered the structure of the APP intracellular domain that since the mutant APPYG protein still undergoes other phosphorylation events. Other products of the amyloidogenic pathway are Aβ40 & 42. Only Aβ40 was detectable and showed a significant 25% decrease in APP^YG/YG^ mice compared to APP^wt/wt^ controls (Fig. 2E), which was also consistent with a decreased β-processing of APP. The change was specific for the Y682G mutation since Aβ40 levels were not changed in the APP^TA/TA^ mice, again in agreement with previous findings [20]. Total APP levels show a 64% increase in APP^YG/YG^ over controls, after normalization with Tubulin (Fig. 2A & B). Whether this indicates an increased total half-life of APP Y682G as compared to WT APP remains to be determined. It is also possible that manipulation of the APP gene locus may have altered the transcription/splicing of the mutant allele. To test for this, we have now performed real-time RT-PCR and the data demonstrate that expression levels of APP mRNA are not affected by the genetic strategy used to do the KI (Fig. 3A). Thus any alterations in APP protein and in its metabolites almost certainly reflect an effect of the YG mutation on the fate of the APP protein, since it is very unlikely that the point mutation will affect the translational efficiency of the KI mRNA. Moreover, this increase in total APP is not always reproducible (APP levels are similar between the two strains in the experiments shown in Fig 2C, Fig. 4 and Fig. 5). The reason for this variability is not presently understood.

Since a- and b-cleavages are mutually exclusive, an increase in one should be compensated by roughly equal decrease in the other if all full length APP is cleaved, in similar proportions, by either a- or b-secretase. However, we report a ∼15-fold increase in soluble sAPP-α but only a ∼3.5 -fold decrease in sAPP-β. Unless *in vivo* sAPP-β is cleared more efficiently than sAPP-α, these differences are not consistent with the aforementioned model. A recent report shows that APP is cleaved in the ectodomain buy an alternative, albeit yet to be identified, protease [21]. In addition, a large fraction of full length APP is processed by lysosomes, presumably after APP is internalized [22] (Fig. 3B). The YG mutation could reduce BACE and lysosomal degradation of APP, if mutant APP has impaired endocytosis [both processes are largely dependent of APP endocytosis [22], [23], [24]). This would also explain the vast increase in a-secretase processing of the YG APP mutant. This hypothesis is presently being investigated.

Also C83 and C99 undergo lysosomal degradation (Fig. 3B), indicating that these APP metabolites are not exclusively cleaved by g-secretase. Interestingly, inhibition of lysosomal degradation results in the appearance of several COOH-terminal APP fragments larger than C99 [see asterisks in Fig. 3B for both primary neurons and primary mouse dermal fibroblasts (MDFs)]. These fragments are either intermediated of APP degradation in lysosomes, or are produced by processing of APP in regions NH_2_-terminal to the BACE1 cutting site, suggesting that the APP ectodomain can be processed by several unknown proteases, in addition to α- and β-secretase.

The decrease in Ab40 level (25%) is not as pronounced as the reduction in sAPPβ levels. This apparent discrepancy can be explained by either reduced clearance of brain Aβ, a compensatory increase in γ-cleavage of C99, or by reduced clearance of C99 by the lysosomes. These possibilities are being investigated.

As noted above, intracellular transport and localization of APP are critical components of APP processing and Aβ production. In fact, a-secretase cleaves mAPP en route to or on the plasma membrane. β-secretase predominantly cleaves mAPP in early endosomes [23], [24] while C99 and C83 are processed by the γ-secretase in endocytic compartments [24]. Thus, the shift toward the non-amyloidogenic processing in APP^YG/YG^ mice may involve a role of Tyr^682^ in trafficking of APP along the secretory pathway. However, the YG mutation neither altered the imAPP/mAPP ratio in brains (Fig. 4A and B) and primary mouse dermal fibroblasts (MDFs) (Fig. 4C), nor changed the levels of plasma membrane mAPP in MDFs (Fig. 3C).

### Normal brain organization and distributions of neural proteins in APP^YG/YG^ mice

A number of studies suggest important neurological roles for APP and APP polypeptides derived by secretases processing. sAPP-α is neuro-protective [25], [26], [27], [28]. A metabolite of sAPP-β interacts with DR6 to trigger neuronal death [29]. Aβ is critical for LTP induction and memory acquisition [30]. AID/AICD modulates cell death, gene transcription and Ca^++^ homeostasis [3], [4], [31], [32], [33], [34], [35], [36], [37], [38], [39], [40]. Because APP derived polypeptides are significantly changed in APP^YG/YG^ mice, we analyzed whether these mutant mice show abnormalities in brain organization. First, a general histopathological examination of the APP^YG/YG^ mouse showed the following. All mice had minimal multifocal myofiber degeneration affecting the appendicular musculature, primarily, the biceps femoris, quadriceps, and triceps brachii muscles. Randomly scattered throughout the striated muscles were small clusters of swollen muscle fibers with increased cytoplasmic eosinophilia and occasional karyorrhexis (Figure 5). These myofibers were surrounded by myocytes with variable cross sectional diameter and occasional rowing of central nuclei interpreted as myofiber regeneration.

**Figure 6:**
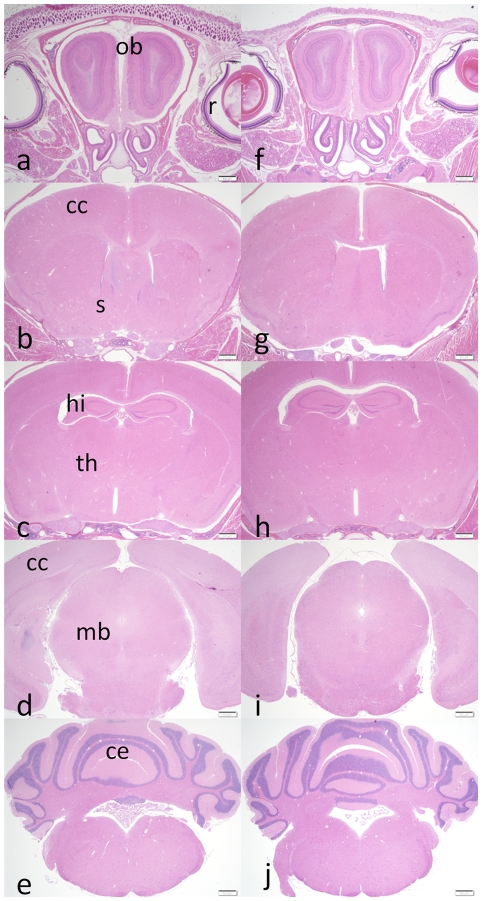
Coronal sections comparing brain anatomy of APP^wt/wt^ with APP^YG/YG^ mice. **A–E** coronal sections of APP^wt/wt^ mouse, **F–J** coronal sections of APP^YG/YG^mouse. **A, F** - Olfactory bulbs. **B, G**- Cerebral cortex. **C, H**- Thalamus. **D, I**- Midbrain. **E, J**- Medulla. Ob-olfactory bulb, r-retina, cc- cerebral cortex, s- striatum, hi- hippocampus, th- thalamus, mb- midbrain, ce- cerebellum.

Coronal step sections of the brain were made at the five following levels- olfactory bulbs, cerebral cortex, thalamus, midbrain and medulla. Each of these regions was further serially sectioned at 250-micron intervals and stained with H&E. Histological examination revealed no structural or anatomical differences between the APP^YG/YG^ and the wild type mice (Fig. 6).

**Figure 7:**
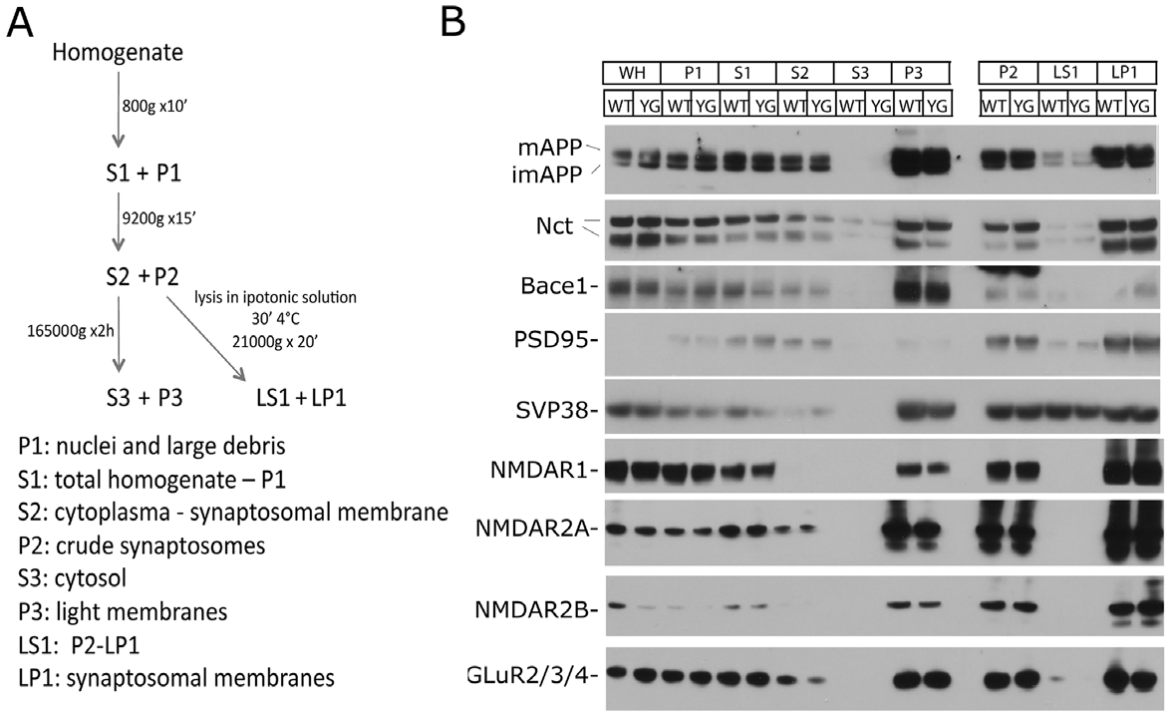
The YG mutation does not alter synaptic proteins. **A**, Schematic representation of synaptosomal preparation. **B**, Synaptic proteins distribution is similar in APP^wt/wt^ and APP^YG/YG^ brains.

Next, we tested distribution of neural proteins using a biochemical approach. Recent evidence suggest a role for APP in synaptic function and numerous data support a role for synaptic dysfunction underlying subtle memory changes in AD [41]. Since the presynaptic regions of neurons are thought to be the main source of Aβ in the brain, attention has been focused on axonal APP trafficking. These studies have unveiled an active role for APP in axonal transport. APP is transported anterogradely by conventional kinesin in tubular vesicles [42], [43], [44], [45], [46], [47], [48]. Although a direct interaction of APP with the motor protein kinesin-1 has been proposed [49], following studies have contradicted this conclusion [50], [51] and shown that APP interacts with kinesin-1 trough the APP-interacting proteins JIP1, a c-Jun N-terminal kinase JNK-signaling scaffold protein [51], [52], [53]. It has also been proposed that β- (BACE1) and γ-secretases transported in APP-containing vesicles and that APP functions as a receptor for the cargo transport [54]. A number of observations suggest that microtubule-dependent axonal transport is impaired in AD human brains [5], [55], [56], [57], [58] as well as APP transgenic mice [5], [59], [60]. Because of the indication that the intracellular domain of APP is important for axonal transport of APP and cargo molecules, we analyzed the synaptic distribution of neural proteins. We studied APP. BACE1, a component of the γ-secretase complex, Nicastrin, and synaptic proteins/receptors, such as PSD95, SVP38, the Glutamate receptors NMDAR1, NMDAR2A, NMDAR2B and GLuR2/3/4. The synaptic levels of these proteins were unchanged (Fig. 7), further supporting the notion that the YG mutation does not affect APP maturation and trafficking and that APP may not be a regulator of fast anterograde axonal transport.

### Absence of APP/Fe65 interaction in APP Y682G mutant mice

**Figure 8:**
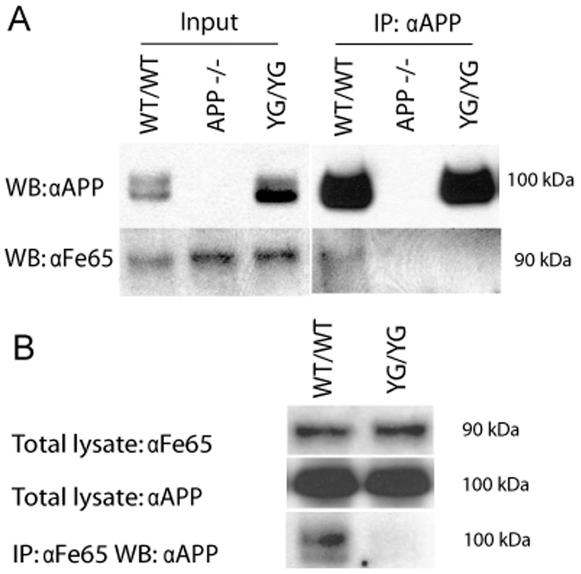
Loss of APP/Fe65 interaction in APP^YG/YG^ mouse brain. **A**, IP of APP from a membrane enriched brain fraction using anti-APP antibody. WB analysis for Fe65 shows an absence of detectable signal in both APP^−/−^ and APP^YG/YG^ compared to APP^wt/wt^. **B**, IP of Fe65 from a membrane enriched brain fraction using anti-Fe65 antibody (I12). WB analysis for APP shows an absence of detectable signal in APP^YG/YG^ mutant mice compared to APP^wt/wt^.

Several cytosolic proteins bind APP. These APP-interacting proteins regulate both APP processing and functions of APP polypeptides *in vitro* [51], [52], [53], [61], [62], [63], [64], [65], [66], [67], [68]. However, the *in vivo* relevance of these findings is still unclear. Most of these interactions involve the YENPTY sequence (amino acids 682–687) of APP. Phosphorylation of APP is consequential. Some proteins interact with APP only when Tyr^682^ is phosphorylated [14], [69], [70], [71]; others, like Fe65, Fe65L1 and Fe65L2, only when this tyrosine is not phosphorylated [13]. The same is true for Thr^668^ [67], [72]. These data suggest that phosphorylation–dephosphorylation on Tyr^682^ and Thr^668^ modulates APP interactions and function. Notably, Tyr^682^ and Thr^668^ phosphorylation is increased in AD brains [9], [73]. The APP/Fe65 interaction is the best characterized of many potential APP/APP C-terminal binding protein complexes. *In vitro* studies have suggested a role for APP/Fe65 complexes in APP metabolism, and for AID-AICD/Fe65 in gene transcription [4], [62], [74]. Tyr^682^ is essential for a robust APP/Fe65 interaction to occur *in vitro* [75] and, as noted above, other *in vitro* evidence shows that Tyr^682^phosphorylation abolishes docking of Fe65 to APP. To establish whether this important role of Tyr^682^ is true *in vivo* co-IP of APP and Fe65 was carried out using brain tissue from APP^YG/YG^, APP^−/−^ and APP^wt/wt^ control mice (Fig. 8). Panel A shows co-IP using α-APP antibody. The loss of detectable signal for Fe65 in both APP^−/−^ and APP^YG/YG^ mice compared to APP^wt/wt^ control clearly demonstrates that Tyr^682^ is specifically required for this interaction to occur. Conversely Panel B shows an IP experiment using α-Fe65 antibody and clearly shows an absence of binding of APP in APP^YG/YG^ mice, again demonstrating the necessity of Tyr^682^ in the APP/Fe65 interaction.

## Discussion

Mis-folding of amyloidogenic Aβ peptides, particularly Aβ42, is a key feature of the AD pathology. APP Y682G mutation in mice clearly results in a large redistribution of APP towards non-amyloidogenic pathway; sAPP-α and C83 are greatly increased while sAPP-β and Aβ40 are decreased (Fig. 2), thus demonstrating the necessary role of the C-terminal in normal activity of the amyloidogenic pathway in the brain and consistent with the results observed *in vitro*. It is not yet apparent why a concurrent reduction in C99 or p-C99 was not observed. It is interesting to speculate, based upon previous findings, how this profound shift in APP processing may influence physiology in APP^YG/YG^ mice. sAPP-α for instance is a proposed growth factor with neuroprotective properties, therefore the 15-fold over expression may result in different growth characteristics and a resistance to stress, although no differences in brain organization were apparent in our analysis (Fig. 5, 6).

A possible mechanism to explain this shift toward the non-amyloidogenic processing may involve the essential role of Tyr^682^ for normal endocytosis of APP as previously shown *in vitro* [11]. A fraction of APP is cleaved by α-secretase in a post-Golgi compartment or at the plasma membrane. Alternatively, some APP is processed by β-secretase in the Golgi or in late endosomes following internalization from the cell membrane. In addition Aβ levels tightly correlate with APP internalization such that Aβ secretion is significantly decreased when APP endocytosis is compromised *in vitro* [23], [24]. However our initial analysis of APP localization shows no difference in cell surface APP (Fig. 4C).

We also demonstrate that Tyr^682^ is necessary for interaction between APP and binding partner Fe65 *in vivo* (Fig. 8). Interestingly Fe65 also plays role in endocytosis of APP. Fe65 simultaneously binds to the cytoplasmic tail of APP and of LRP1 into a trimeric complex [62], [76], [77]. This interaction results in accelerated endocytosis of APP via clathrin-coated pits and in delivery to late endosomal compartments for cleavage by β- and γ-secretase to generate Aβ [72]. The decrease in amyloidogenic processing in APP^YG/YG^ mice may be consistent with this data. Another known function of the APP/Fe65 interaction, which should be absent in APP^YG/YG^ mice, is the transcriptional activity of AID/Fe65/Tip60 complex [4], although further investigation will be needed to determine if this is true. Also of note is that phosphorylation of Tyr^682^ also disrupts interaction of APP with Fe65 and other PTB domain proteins [13], [14] and this may be one commonality between APP^YG/YG^ mice and AD brain.

A comparative analysis between APP^YG/YG^, APP^TA/TA^ and control mice clearly indicates steady-state APP phosphorylation predominantly occurs on Thr^668^ and not Tyr^682^ in normal brain (Fig. 2D). Counter intuitively, this observation may exemplify the importance of Tyr^682^ phosphorylation. Evidence shows Tyr^682^ is hyperphosphorylated in the AD Brain [9], [10]. It is plausible that excessive phosphorylation at this functionally important residue could lead to toxic effects, given its relative scarcity under normal conditions. For example, if Tyr^682^ phosphorylation plays a role in targeting APP for degradation via secretase, lysosomal or proteasomal pathways, it would explain why detection of pY682-APP and pY682-APP-CTFs proteins would be very difficult in a normal brain.

Given the functional redundancy provided by APLP1 and APLP2 much of the phenotype in APP^YG/YG^ mice is potentially masked. Therefore a very important goal is to cross APP^YG/YG^ mice with APLP2^−/−^ mice. If significant APP functionality is facilitated by Tyr^682^ then we may see aspects of the severe APP^−/−^/APLP2^−/−^ mice phenotype reproduced in a context that allows a much more detailed picture of APP function to be dissected. Physiological characterization of this model in addition to APP^YG/YG^ mice is therefore of great interest.

In summary, we have successfully generated two APP KI mouse lines, APP Y682G & T668A, and carried out an initial characterization focusing on APP Y682G. Both APP processing and the APP/Fe65 interaction are significantly altered as a result of this mutation, in agreement with previous *in vitro* studies. In addition these findings have a therapeutic implication, demonstrating that manipulation of this amino acid could increase production of the sAPPα, considered a protective protein, and decrease the generation of toxic fragments such as Aβ40 & 42. without negative physiological consequences. Overall, the data suggests a very important *in vivo* role for Tyr^682^ in the brain.
